# Supplementary material for: Differences in the Concentration of Anti-SARS-CoV-2 IgG Antibodies Post-COVID-19 Recovery or Post-Vaccination
Source: Cells. 2021 Jul 31;10(8):1952. doi: 10.3390/cells10081952 (PMC8391384; doi:10.3390/cells10081952)
Supplement: Supplementary file 1 [file cells-10-01952-s001.zip › cells-1210790-supplementary.pdf]

Table S1. Detailed characteristics of the sex and age of the study participants, taking into account the division into subgroups

SARS-CoV-2 and vaccination status	N	Female					Male				
		Age category (years)									
		<=35	36-45	46-55	56-65	65+	<=35	36-45	46-55	56-65	65+
**uninfected, unvaccinated**	**24**	4	2	3	5	0	2	4	2	1	1
**asymptomatic SARS-recovered, unvaccinated**	**15**	2	0	2	1	1	2	1	4	2	0
**symptomatic SARS-recovered, unvaccinated**	**82**	11	14	12	15	6	2	6	4	5	7
**2 weeks after the first vaccine dose, uninfected**	**8**	2	1	1	0	1	0	1	0	1	1
**3 week safter the first vaccine dose,uninfected**	**11**	4	1	0	2	1	0	1	0	1	1
**3 weeks after the first vaccine dose, SARS-recovered**	**9**	1	1	3	3	0	0	0	0	0	1
**1 week after the second vaccine dose, uninfected**	**17**	4	1	4	2	2	1	2	0	1	0
**1 week after the second vaccine dose, SARS-recovered**	**10**	1	2	2	3	0	0	0	0	0	2
**2 weeks after the second vaccine dose, uninfected**	**39**	7	3	6	7	7	1	1	1	3	3
**2 weeks after the second vaccine dose, SARS-recovered**	**22**	3	4	4	3	4	1	0	0	0	3
**3 weeks after the second vaccine dose, uninfected**	**24**	5	4	4	1	2	1	2	2	2	1
**3 weeks after the second vaccine dose, SARS-recovered**	**36**	3	14	7	7	1	2	1	1	0	0
**4 weeks after the second vaccine dose, uninfected**	**55**	7	12	16	9	2	1	3	1	2	2
**4 weeks after the second vaccine dose, SARS-recovered**	**31**	6	10	3	5	1	2	3	1	0	0
**5 weeks after the second vaccine dose, uninfected**	**68**	9	18	10	17	0	1	4	5	3	1
**5 weeks after the second vaccine dose, SARS-recovered**	**26**	1	5	7	6	2	1	0	1	3	0
**Total**	**477**	**70**	**92**	**84**	**86**	**30**	**17**	**29**	**22**	**24**	**23**

Figure S1. Analysis of the mean concentrations of G class antibodies against the S1 subunit of SARS-CoV-2 in the SARS-recovered and vaccinated subgroups

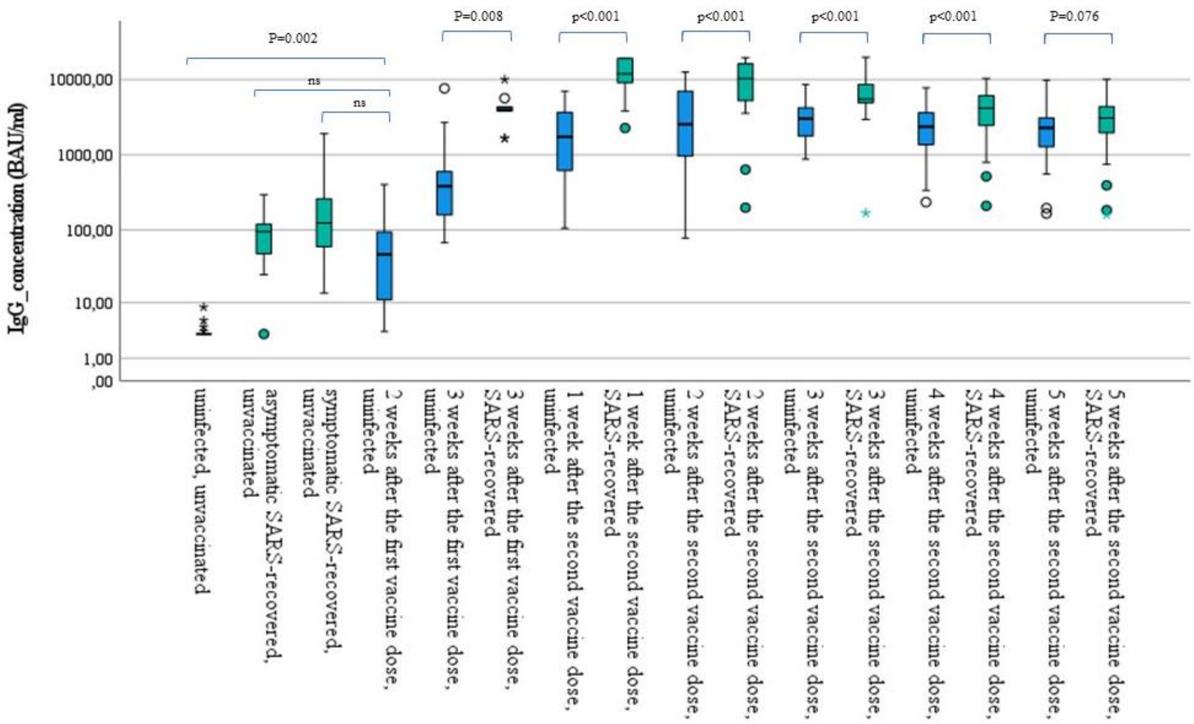

Comparisons of antibody levels between the analysed patients subgroups. Blue colour indicates the concentration of anti-SARS-CoV-2 IgG antibodies in seronegative individuals before vaccination. Individuals with confirmed SARS-CoV-2 infection prior to receiving the mRNA vaccine dose, as well as seropositive unvaccinated individuals, are marked in green.

Tests of Homogeneity of Variances

		Levene Statistic	df1	df2	Sig.
IgG_concentration (BAU/ml)	Based on Mean	22,709	15	461	,000
	Based on Median	15,241	15	461	,000
	Based on Median and with adjusted df	15,241	15	164,019	,000
	Based on trimmed mean	21,057	15	461	,000

ANOVA

IgG_concentration (BAU/ml)

	Sum of Squares	df	Mean Square	F	Sig.
Between Groups	3908014005,181	15	260534267,012	35,305	,000
Within Groups	3402009785,832	461	7379630,772		
Total	7310023791,012	476			

Figure S2. Means Plots for convalescent and vaccinated subgroups.

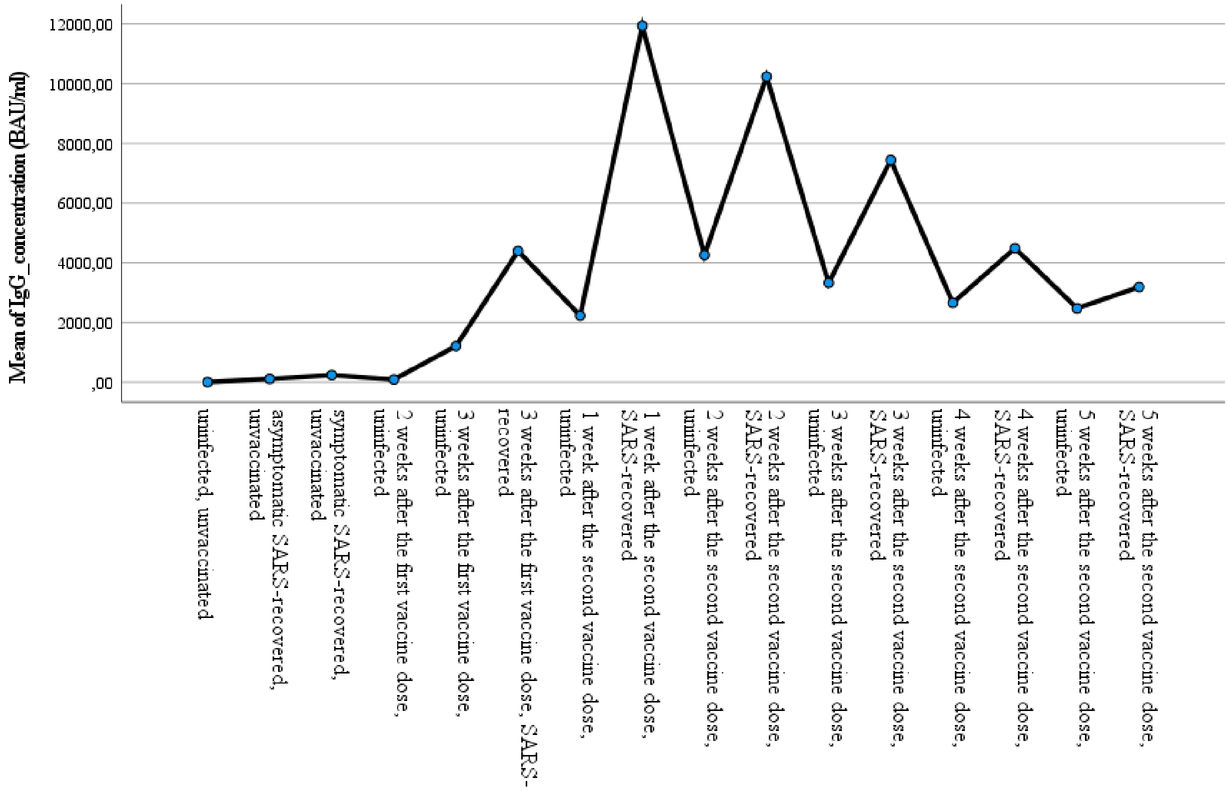

Table S2. One-way ANOVA for comparisons between subgroups

	Sum of Squares	df	Mean Square	F	Sig.
Between Groups	46242,329	1	46242,329	11,214	0,002
Within Groups	123707,811	30	4123,594		
Total	169950,140	31			

	Sum of Squares	df	Mean Square	F	Sig.
Between Groups	2351,804	1	2351,804	0,208	0,653
Within Groups	237991,400	21	11332,924		
Total	240343,204	22			

	Sum of Squares	df	Mean Square	F	Sig.
Between Groups	503047953,900	1	503047953,900	22,496	0,000
Within Groups	1319339964,442	59	22361694,313		
Total	1822387918,342	60			

	Sum of Squares	df	Mean Square	F	Sig.
Between Groups	593860671,424	1	593860671,424	37,843	0,000
Within Groups	392319196,395	25	15692767,856		
Total	986179867,819	26			

	Sum of Squares	df	Mean Square	F	Sig.
Between Groups	255855376,104	2	127927688,052	8,277	0,001
Within Groups	911932802,458	59	15456488,177		
Total	1167788178,562	61			

	Sum of Squares	df	Mean Square	F	Sig.
Between Groups	67422256,608	1	67422256,608	14,115	0,000
Within Groups	391675211,452	82	4776526,969		
Total	459097468,060	83			

	Sum of Squares	df	Mean Square	F	Sig.
Between Groups	9643545,062	1	9643545,062	3,213	0,076
Within Groups	276125620,968	92	3001365,445		
Total	285769166,030	93			

Table S3. Mean anti-SARS-CoV-2 IgG concentrations by gender and age of the study participants.

	**Female**					**Male**				
	**<= 35**	**36 – 45**	**46 - 55**	**56 – 64**	**65+**	**<= 35**	**36 - 45**	**46 - 55**	**56 - 64**	**65+**
**uninfected, unvaccinated**	3.20	3.20	5.32	3.27	.	4.29	3.20	3.20	3.20	3.20
**asymptomatic SARS-recovered, unvaccinated**	57.43	.	59.53	94.95	104.32	45.41	115.10	233.41	58.09	.
**symptomatic SARS-recovered, unvaccinated**	113.49	121.95	287.37	437.77	200.30	101.42	136.60	88.26	155.35	438.51
**2 weeks after the first vaccine dose, uninfected**	58.83	131.04	43.79	.	3.55	.	4.32	.	26.08	404.45
**3 weeks after the first vaccine dose,uninfected**	375.49	945.10	.	5192.90	83.82	.	67.62	.	148.40	170.72
**3 weeks after the first vaccine dose, SARS-recovered**	5685.00	1666.00	3335.67	6125.00	.	.	.	.	.	3840.00
**1 week after the second vaccine dose, uninfected**	2117.15	1747.20	1251.96	3652.10	2316.65	4449.00	2112.00	.	2050.90	.
**1 week after the second vaccine dose, SARS-recovered**	9164.00	14182.00	10740.15	14382.17	.	.	.	.	.	8624.25
**2 weeks after the second vaccine dose, uninfected**	7460.89	8190.83	6441.80	2970.64	2285.33	2010.00	248.00	2354.00	1528.90	1580.67
**2 weeks after the second vaccine dose, SARS-recovered**	8126.67	9335.13	12698.25	10439.00	10290.17	3840.00	.	.	.	12160.00
**3 weeks after the second vaccine dose, uninfected**	4799.08	4772.90	2818.60	2929.80	1367.50	2015.50	3836.80	2521.35	1368.10	2369.00
**3 weeks after the second vaccine dose, SARS-recovered**	4203.33	10009.61	5087.21	7463.86	5376.00	4022.90	8649.50	5255.50	.	.
**4 weeks after the second vaccine dose, uninfected**	3223.93	3209.92	2484.94	1779.22	2260.75	2506.00	4119.67	1056.50	3264.50	1193.50
**4 weeks after the second vaccine dose, SARS-recovered**	5575.08	5218.65	3249.83	2165.00	7926.50	5406.25	3062.67	5076.50	.	.
**5 weeks after the second vaccine dose, uninfected**	2549.78	2515.36	2332.80	2448.29	.	1094.50	2451.63	3254.10	2275.00	1093.50
**5 weeks after the second vaccine dose, SARS-recovered**	3333.50	2255.25	2968.57	3915.75	2338.25	4809.50	.	3301.50	3760.50	.
